# Understanding consumer acceptance of intervention strategies for healthy food choices: a qualitative study

**DOI:** 10.1186/1471-2458-13-1073

**Published:** 2013-11-13

**Authors:** Colin Bos, Ivo A Van der Lans, Frank J Van Rijnsoever, Hans CM Van Trijp

**Affiliations:** 1 Marketing and Consumer Behaviour Group, Wageningen University, Hollandseweg 1, Wageningen 6706 KN, the Netherlands; 2 Innovation Studies, Copernicus Institute of Sustainable Development, Utrecht University, Heidelberglaan 2, Utrecht 3584 CS, the Netherlands

**Keywords:** Public health, Consumer acceptance, Intervention strategies, Low-calorie, Food choice, Obesity prevention

## Abstract

**Background:**

The increasing prevalence of overweight and obesity poses a major threat to public health. Intervention strategies for healthy food choices potentially reduce obesity rates. Reviews of the effectiveness of interventions, however, show mixed results. To maximise effectiveness, interventions need to be accepted by consumers. The aim of the present study is to explore consumer acceptance of intervention strategies for low-calorie food choices. Beliefs that are associated with consumer acceptance are identified.

**Methods:**

Data was collected in the Netherlands in 8 semi-structured interviews and 4 focus group discussions (N = 39). Nine archetypical strategies representing educational, marketing and legal interventions served as reference points. Verbatim transcriptions were coded both inductively and deductively with the framework approach.

**Results:**

We found that three beliefs are related to consumer acceptance: 1) general beliefs regarding obesity, such as who is responsible for food choice; 2) the perceived effectiveness of interventions; and 3) the perceived fairness of interventions. Furthermore, the different aspects underlying these general and intervention-specific beliefs were identified.

**Conclusions:**

General and intervention-specific beliefs are associated with consumer acceptance of interventions for low-calorie food choices. Policymakers in the food domain can use the findings to negotiate the development of interventions and to assess the feasibility of interventions. With respect to future research, we recommend that segments of consumers based on perceptions of intervention strategies are identified.

## Background

From 1980 to 2008, the overweight and obese population almost doubled worldwide. It now consists of an estimated 1.46 billion adults and 170 million children [1]. People with overweight and obesity are more vulnerable to non-communicable diseases such as type II diabetes, cancer, and cardiovascular diseases [2]. The costs of these diseases in terms of quality of life and healthcare are enormous. The medical expenditure associated with obese individuals is estimated at 30 per cent higher than normal-weight peers [3]. Thus, overweight and obesity pose both personal and public health concerns.

The growth of obesity rates is the result of a systematic energy imbalance primarily due to excessive intake of calories [4]. Although (the lack of) physical activity is an important part of the obesity problem, curtailment of overconsumption is of greater importance. Limiting calorie intake directly affects weight status and also adds to the impact of physical activity on weight status [5]. Intervention strategies that reduce the amount of calories consumers choose potentially achieve both economic benefits and improvements of personal and public health [6].

A broad array of intervention strategies, varying from public health campaigns to the taxation of high-calorie foods, has been implemented to reduce obesity prevalence [7]. However, recent systematic reviews concerning the effectiveness of intervention strategies show mixed results. A large structured analysis of policy interventions [7] reveals inconclusive results about the behavioural impact of different types of interventions. Other reviews also claim that for many interventions only limited evidence of their effectiveness can be found [8-11].

To increase the effectiveness of interventions, Andreasen [12] argues that they need to be consumer-driven. More specifically, intervention strategies should be based on an understanding of consumers’ experiences, values, and needs, which jointly accumulate into consumer acceptance of intervention strategies. The term ‘consumer acceptance’ is used throughout the article because the interventions of interest target food choice (consumption) behaviour. It is important to note that the present study’s method allowed for assessing these interventions from a consumer point of view as well as from a broader, social perspective.

The degree of consumer acceptance affects both the effectiveness and the implementability of interventions. Reactance theory [13] suggests that low levels of acceptance towards an intervention cause consumers to adopt or strengthen an attitude that is contrary to the desired behaviour, thereby increasing resistance to perform the desired behaviour. In contrast, a high level of acceptance elicits rationalization, causing consumers to be more likely to approve of interventions and to adopt the intended behaviour [14]. In addition, the acceptability of an intervention is an important condition for its implementation. Stakeholders will be reluctant to intervene without public support [15]. Knowledge of factors influencing consumer acceptance of intervention strategies thus is crucial. Within the food domain, a number of surveys have found that beliefs about the causes of obesity affect support for obesity prevention policies [16-18]. However, a structured insight into consumers’ perceptions of interventions is lacking [19].

The present study aims to fill this gap by exploring not only consumer acceptance, but also the perceived effectiveness and the perceived fairness of intervention strategies for low-calorie food choices. A qualitative approach is adopted to identify concepts and processes at the individual level that will enrich the dominant quantitative focus on the effectiveness of interventions. The present study uses both social marketing theory and existing research on consumer acceptance of interventions as a theoretical framework. The results provide guidance for the assessment of consumer acceptance of (future) interventions in the food domain.

### Theoretical framework

### Social marketing

Social marketing uses commercial marketing concepts such as market segmentation and consumer research to achieve social change [20]. A growing body of research claims that social marketing provides a promising framework for improving health both at the individual level and the wider societal level [21]. A significant part of social marketing is to understand consumers before and at the outset of interventions [12].

**Figure 1:**
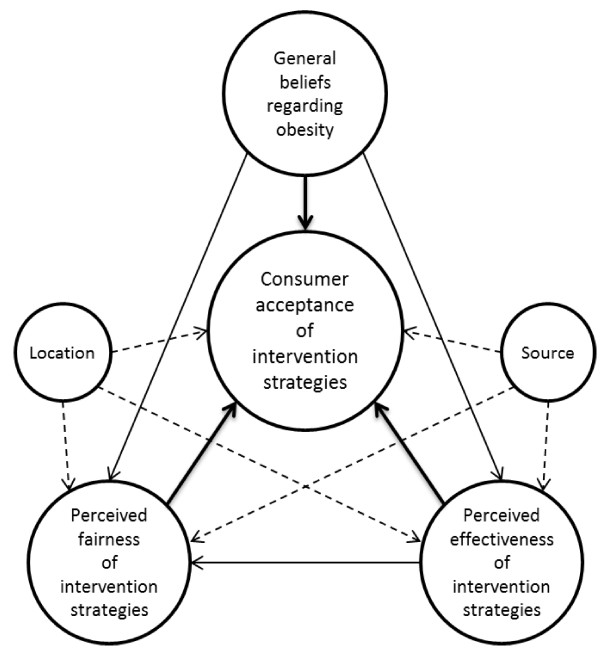
**Schematic representation of the research framework.** Continuous lines: relations of interest that are found in the environmental domain. Dotted lines: additional relations of interest.

To achieve this understanding for intervention strategies in the food domain, learnings are extracted from the environmental domain (e.g. car use vs. public transport), where acceptance of interventions has been studied more extensively [22-24]. Within that domain, general beliefs and intervention-specific beliefs have proven to affect acceptance of interventions. General beliefs reflect one’s overall orientations, opinions and attitudes towards a particular public issue. An example of a general belief in the environmental domain is one’s perception of responsibility for traffic congestion. In addition, two intervention-specific beliefs emerge from that body of literature: the perceived effectiveness and the perceived fairness of interventions [22]. The perceived effectiveness refers to whether an individual believes that an intervention will actually lead to the intended behaviour; therefore it does not necessarily reflect an intervention’s actual effectiveness. Likewise, the perceived fairness relates to an individual’s belief that the implementation of a specific intervention is a fair way to stimulate the intended behaviour. Our research framework (Figure 1) serves to identify both the general and intervention-specific beliefs for the food domain.

#### Types of intervention strategies

Various classifications of intervention strategies for public health issues exist [7,25-27]. Although these classifications share similarities, most of them are strongly policy-oriented. Among them, Rothschild’s social marketing framework allows for the exploration of both policy and non-policy oriented intervention strategies.

Rothschild distinguishes between three types of tools for intervention strategies in public health issues: education, marketing and law. These tools differ on the basis of their reinforcement/reward and degree of voluntary change. Education refers to voluntary adaptation of behaviour by providing information to consumers. Marketing also refers to voluntary adaptation of behaviour; however it does so by reinforcing consumers. Law refers to non-voluntary adaptation of behaviour by using coercion and by punishing consumers for non-compliance. The present study uses nine archetypical interventions based on these three tools. The archetypes and matching examples are adapted from Van Trijp et al. [28] and can be found in Table 1.

**Table 1:** List of archetypical intervention strategies and corresponding examples

**Stimuli** ***Interventions***	***Example***
	**Law**
Making unhealthier products more expensive	An increase of taxes on high-calorie products
Making healthier products less expensive	A decrease of taxes on low-calorie products
Restricting the promotion of unhealthier products	Prohibition of promotion of high-calorie products at bus shelters
	**Marketing**
Promoting healthier products	Promotion of a low-calorie product by a famous athlete on behalf of the food supplier
Decreasing the accessibility of unhealthier products	Placement of high-calorie products on the bottom shelf and low-calorie products at eyesight in a supermarket
Increasing the availability of healthier products	Provision of low-calorie alternatives for high-calorie products by food suppliers
	**Education**
Providing calorie information of personal choices in relation to choices of others	Use of a receipt that indicates the amount of calories one has bought and the amount others buy, implemented in a canteen by the employer
Providing food labels with calorie information	Provision of extensive traffic-light labels on food products by food suppliers
Providing information about healthier eating habits	Provision of information about how to create low-calorie eating habits through a governmental campaign

In addition to distinguishing between education, marketing, and law, our framework also explores the effects of both the intervention’s physical location (e.g. restaurants and schools) and the intervention’s source/manager (e.g. the government and the food industry) of an intervention on consumer acceptance. Among the spectrum of interventions, consumer acceptance likely differs due to the different implications that combinations of tools, locations, and sources of interventions have for consumers [29].

## Methods

### Ethical approval

The present study’s interview and focus group protocol was submitted to the Social Science Ethics Committee of the Wageningen University and subsequently approved for fulfilling the Wageningen University code of conduct.

### Design and participants

Data was collected in the Netherlands in two rounds. The first round consisted of eight semi-structured interviews with an average duration of 50 minutes and the second round used four focus group discussions (6–9 participants), each 2 hours long. The interviews gained individual views on the concept of acceptability, while the focus groups captured the dynamics and the range of the concept of acceptability by allowing participants to build on and react to responses of others [30]. The interviews also provided the possibility to make procedural adjustments for the focus groups when needed.

Participants were recruited through a recruitment agency, which uses its own panel. Selection of participants aimed at a sufficiently heterogeneous sample in terms of gender, age and income level (Table 2). A written informed consent was obtained from participants before the start of the interviews and focus groups. For their participation, participants received the standard monetary compensation, according to the recruitment agency’s policy. Both the interviews and the focus groups were held at the recruitment agency’s facilities and were conducted by the first author. During the focus groups, an observer was present to take notes and to ensure all items in the guide were addressed.

**Table 2:** Sample characteristics

	***Interviews*** ***N = 8***	***Focus groups*** ***N = 31***
*Gender*		
Male	5	14
Female	3	17
*Age*		
18-35	3	13
35-50	3	10
50-65	2	8
*Income level*		
Less than modal	3	10
Approximately modal	3	14
Higher than modal	2	7

### Stimuli and materials

The stimulus material consisted of eight archetypical intervention strategies (Table 1). Each intervention was presented on a separate A5 size paper following a standardised format with three blocks of information. The first block gave a definition of the archetypical intervention, e.g. “Making unhealthier product more expensive”. The second block provided a detailed example of the archetype that more firmly categorised the interventions in terms of education, marketing, and law: “An increase of taxes on high-calorie products”. The third block was used to provide an image of the detailed example.

In addition to the eight interventions used in the interviews, one extra intervention was introduced during the focus groups. Based on remarks during the interviews, “Restricting the promotion of unhealthier products” was added as a ninth intervention. Half of the interview participants expressed the opinion that excessive promotion of high-calorie foods was a significant contributor to the obesity problem. Some therefore suggested adding an intervention that restricted such promotion to the other archetypical interventions.

### Procedure

The interviews were divided into five parts (Table 3). After a short introduction, the interviewer explicitly invited and encouraged participants to voice their personal opinions and stressed that there were no correct or wrong answers. As a warm-up, participants were asked questions about their interpretation of (un)healthy food choices and the perceived responsibility for food choices. Following the warm-up, the interviewer explained that in the context of the interview, the interpretation of a healthy choice was restricted to a choice relatively low in calories, whereas an unhealthy choice was restricted to a choice with a relatively high caloric value. This interpretation was used to avoid that participants would define healthy choices at different levels of abstraction [31].

**Table 3:** Interview and focus group topics

**Procedure** ***Topic***	***Content***
Part 1: Introduction	Consent form and word of welcome
Healthy and unhealthy choices	What, in your opinion, is a(n) (un)healthy food choice?
	Do you find it easy or hard to make healthy food choices?
Responsibility for food choices	Who is responsible for the healthiness of the food choices you make?
Part 2: Acceptability of intervention strategies	Do you think changes are warranted to stimulate low-calorie food choices?
**(Introduction of the archetypical intervention strategies)**	Do you think the archetypical interventions are acceptable, when their goal is to stimulate you to make low-calorie food choices?
Part 3: Perceived effectiveness of intervention strategies	Do you think the archetypical interventions will lead you to make low-calorie food choices?
Part 4: Perceived fairness of intervention strategies	Do you think the archetypical interventions are a fair way to stimulate you to make low-calorie food choices?
Part 5: Acceptability of intervention strategies (2)	Do you think the archetypical interventions are acceptable, when their goal is to stimulate you to make low-calorie food choices?
Wrap-up	Explanation of the research context and a word of thanks

To explore participants’ initial attitude towards interventions, the interviewer asked them whether they thought changes were needed to stimulate healthy food choices. Subsequently, the interviewer introduced the archetypical interventions one by one and in a fixed order. Next, participants were asked the interview’s main question: “Do you think these interventions are acceptable, when their goal is to stimulate you to make low-calorie food choices?” The interviewer instructed participants to individually sort the interventions into three groups: Acceptable, Neutral/don’t know, and Not acceptable. After the sorting task, the interviewer asked participants to elaborate on their classification. The interviewer then asked if and how each archetypical intervention could become more and/or less acceptable. Through the use of prompts participants were also asked whether the source and the location of an intervention influenced its acceptability (e.g. “Do you think this intervention would be more/less acceptable if it was executed by another source?” and “Do you think this intervention would be more/less acceptable at other locations?”). Last, the interviewer asked participants whether they thought the interventions were acceptable for others and/or certain groups. Part 2 took up about half of the time of both the interviews (± 25 minutes) and the focus groups (± 55 minutes).

After a 5-minute break, the procedure for Part 2 was repeated for the perceived effectiveness (Part 3) and the perceived fairness (Part 4) of the archetypical interventions. For these parts the main questions were “Do you think the archetypical interventions will lead you to make low-calorie food choices?” and “Do you think the archetypical interventions are a fair way to stimulate you to make low-calorie food choices?”, respectively. However, upon completion of the individual sorting task, a smaller number of interventions were addressed during these parts of the discussion due to time constraints. The moderator did ensure that all tools (education, marketing, and law) were covered.

During Part 5 participants were instructed once more to sort the interventions on the basis of their acceptability, without the opportunity to refer to the initial classification of Part 2. Differences between the classifications in Part 2 and Part 5 were then identified and participants were given the opportunity to elaborate on any changes they made. Last, the interviewer explained the context of the study and participants were given the opportunity to remark on the interview. A word of thanks and the distribution of a monetary compensation concluded each session.

Because no methodological issues emerged during the interviews, the same procedure was used for the focus group discussions. The moderator also used the same questions and stimuli materials for the discussions (except for one additional intervention scenario).

A noteworthy finding is that during the opportunity to give remarks, participants more than once declared that they were pleased to have been part of such a discussion. They particularly appreciated the opportunity to voice their own opinions and to discuss public health issues with each other. Therefore, there is little reason to believe that social desirability influenced the legitimacy of participants’ responses.

### Data analysis

The semi-structured interviews and focus group discussions were recorded and transcribed verbatim by the first author and two assistants. Atlas.ti 6.2 (Atlas.ti Scientific Software Development GmbH, Berlin, Germany) was used to carry out the content analysis. To analyse the data, the framework approach was used [32]. This means that after familiarisation with the data by listening to the audio, watching video footage and reading the transcripts, dominant themes were extracted both deductively and inductively. The deductive part consisted of identifying quotes and aspects that fitted the beliefs of the research framework. The inductive part of the coding consisted of identifying additional themes and aspects that emerged from the data itself.

The interview transcripts were analysed and coded first, resulting in an initial codebook. Subsequently, this codebook was used to analyse the focus group transcripts while leaving room for new codes to emerge. During the analysis of the last focus group transcript no new codes emerged, suggesting that theoretical saturation was reached.

## Results

The results are discussed around the three beliefs that were part of the research framework: general beliefs regarding obesity, the perceived effectiveness of interventions, and the perceived fairness of interventions. Table 4 displays the three beliefs and the underlying aspects that emerged. The table also gives an overview of the amount of quotes that were given about these aspects during the different parts of the interviews and focus groups. If applicable, the results section distinguishes between education, marketing, and legal interventions.

**Table 4:** Amount of quotes given about aspects of the general and intervention-specific beliefs in the eight interviews (I) and the four focus groups (FG)

	**Part 2 acceptability**		**Part 3 effectiveness**		**Part 4 fairness**		**Part 5 acceptability 2**	
	**I**	**FG**	**I**	**FG**	**I**	**FG**	**I**	**FG**
**1. General beliefs regarding obesity**								
*1.1 Responsibility for food choice*	7	9	4	4	0	11	3	6
*1.2 Problem awareness*	8	14	0	2	3	10	1	2
**2. Perceived effectiveness of interventions**								
*2.1 Perceived personal effectiveness* &	8	14	0	2	3	10	1	2
*Perceived societal effectiveness*	13	13	25	21	7	9	0	0
*2.2 Effectiveness other domains*	6	5	8	4	3	5	1	1
*2.3 Accessibility of low-calorie products*	7	10	2	5	1	9	0	0
*2.4 Healthiness of low-calorie products*	4	13	2	8	4	5	0	0
*2.5 Identifiability low-calorie products*	8	12	4	19	5	2	0	0
*2.6 Combinations of interventions*	7	10	4	3	4	2	0	1
**3. Perceived fairness of interventions**								
*3.1 Encouragement vs. discouragement*	11	11	0	3	5	11	0	0
*3.2 Societal fairness*	5	12	0	2	4	6	0	3
*3.3 Effects on consumer groups*	5	14	4	3	2	2	2	2
*3.4 Effects on food industry*	3	8	2	2	1	2	1	0
*3.5 Fairness of disseminated information*	24	23	5	17	5	4	0	5
*3.6 Liberty, autonomy, and privacy*	5	6	1	8	11	23	4	2
**Source**	8	16	8	11	5	5	0	2
**Location**	22	13	6	10	5	14	0	5

For illustration purposes, several quotes are given. These quotes, which characterise the different beliefs and aspects, come from different parts of the interviews and discussions (for example: a quote regarding the effectiveness of an intervention could have been given during a discussion about the acceptability of that intervention). Furthermore, results from the interviews and the focus groups are discussed jointly because no differences existed with respect to the beliefs and underlying aspects that emerged.

### Consumer acceptance of intervention strategies for low-calorie food choices

**Figure 2:**
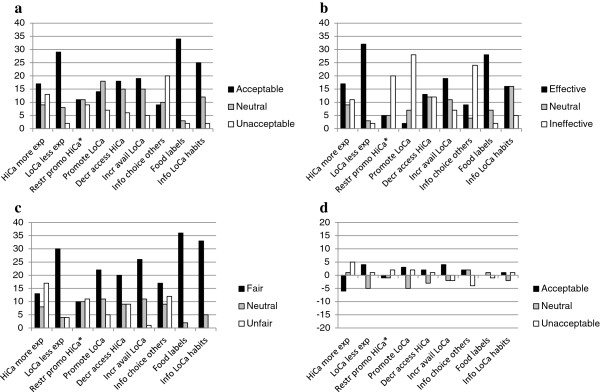
**An overview of participants’ ratings.** Acceptability **(a)**, perceived effectiveness **(b)**, and perceived fairness **(c)** of interventions. Differences between the first and second ratings of acceptance are depicted in **(d)**. *’Restricting promotion of high-calorie foods’ was added after the interviews and therefore has eight ratings less.

Figure 2a depicts the acceptability of the nine archetypical interventions. When participants were asked to elaborate on the acceptability of interventions in Part 2, beliefs about both the effectiveness and the fairness of interventions frequently came up. Because these intervention-specific beliefs were also addressed individually in Part 3 and Part 4, respectively, they will be discussed separately in further parts of the results section.

In addition to intervention-specific beliefs, participants also often expressed general beliefs regarding the obesity problem while elaborating on the acceptability of interventions. The next section gives an overview of the themes that can be discerned.

### General beliefs regarding obesity

Two dominant themes emerged with respect to general beliefs regarding obesity: responsibility for food choice and problem awareness.

#### Responsibility for food choice

A first theme that emerged was the responsibility for food choice. Some participants expressed that, above all, an individual is personally responsible for food choice. Therefore they expressed less acceptance of being stimulated to choose low-calorie foods through interventions:

"I think I'm responsible for what I buy. (…) What gets in my house and what I eat, as a consumer I'm responsible for that. Who is someone else to tell me what to choose?"

Although virtually all participants acknowledged consumers' own responsibility for making food choices, a large number also pointed at the additional responsibility of other stakeholders such as the government and food suppliers. Many of these participants expressed higher acceptance of interventions, especially strategies based on education and the marketing of low-calorie food products. As one man stated:

"I think that marketing of food suppliers certainly has a big influence (on consumer choice). The way they approach consumers; I think they do have a responsibility to help consumers choose healthily".

Participants also frequently mentioned parents’ responsibility for the food choices of their children. All agreed that parents are fully responsible for the healthiness of their children’s food choices and therefore mentioned mothers and fathers as most acceptable sources for interventions. Within the scope of parental interventions, both education about the healthiness of foods and the marketing of healthy choices at home were perceived as most acceptable.

#### Problem awareness

In addition to responsibility for food choice, problem awareness emerged as a second general belief that was related to the acceptance of interventions. Participants recognised that obesity rates have risen during the last decades. Most of the participants attributed this increase to a food environment that promotes overeating. The main characteristics of such an environment consisted of both the availability of high-calorie fast-foods and the relative price advantage of high-calorie foods over low-calorie foods. A majority of participants felt that interventions generally were acceptable if they improved the healthfulness of the environment. A minority disagreed, however, and expressed less acceptance of interventions by claiming that the current food environment provides enough opportunity to maintain a healthy lifestyle.

Another issue that contributed to the obesity problem was the indistinctness of healthy and unhealthy choices. Participants regularly expressed dissatisfaction with opposing claims regarding food products in the media. The resulting ambiguity impaired the ability to make healthy choices. As an older man put it:

“In the 70’s they (the government) promoted certain fats. But they stopped doing that because it wasn’t healthy. Currently, it’s the same with milk: some say it’s good for you, other say it’s not. How do I know what is true?”

### Perceived effectiveness of intervention strategies for low-calorie food choices

The upcoming section will describe the aspects that emerged with regard to the perceived effectiveness of interventions. Ratings of the perceived effectiveness of the archetypical interventions are depicted in Figure 2b.

#### Perceived personal and societal effectiveness of interventions

Participants often distinguished between how effective they perceived interventions to be for themselves and for society as a whole. While some participants thought that the effectiveness of interventions was fairly equal on a personal and societal level, others did not.

Participants who did perceive differences in effectiveness particularly pointed at educational and marketing interventions. They stated that these interventions would not be effective for them personally, but they would be on a societal level. A woman claimed:

“For me this (traffic-light labeling) would not lead to more low-calorie choices. But I do think that others who are not aware of nutritional values would choose low-calorie more often because of these labels.”

#### Perceived effectiveness of similar interventions in other domains

To assess the effectiveness of interventions for low-calorie food choices, participants often relied on their personal knowledge about the effectiveness of tobacco-, alcohol- and transport interventions. They used this knowledge to infer the effectiveness of similar interventions in the food domain. Referring to the domain of tobacco, a majority of participants considered marketing interventions that decrease the accessibility of high-calorie foods to be effective:

“You can eat unhealthy everywhere currently, just like you could smoke everywhere in the past. Nowadays that’s different; there’s an immense pressure on smoking and for that reason I see less people smoke. That could happen with unhealthy foods as well”.

Using a similar analogy, a few participants claimed that educational interventions are ineffective by pointing at a lack of success in other domains. Referring to on-pack nutrition labels, a current smoker stated:

“It's like with cigarettes, the box contains warnings as ‘Smoking is unhealthy’ and ‘Smoking kills’, but everyone continues smoking”.

#### Accessibility of low-calorie products

Participants furthermore stressed that interventions need to make low-calorie choices accessible both physically and financially to be effective. Some participants reported that they knowingly make fewer healthy choices because of the relatively high price of such choices. To increase effectiveness, marketing and legal interventions should therefore bridge the gap between prices of cheap high-calorie foods and more expensive low-calorie foods. As a female student explained:

“As a student I regularly choose French fries, because they only cost €1.50. If I want to eat a healthy sandwich, that will cost me almost three times as much! If it was equally expensive, I would choose more healthily.”

Similar worries were expressed concerning the physical accessibility of low-calorie food choices. In line with the aforementioned food environment that promotes overeating, participants thought that high-calorie choices were more accessible than low-calorie choices. A majority therefore stated that interventions should make low-calorie choices easier to obtain physically. Some participants saw an opportunity for the food supply to provide (new) low-calorie versions of high-calorie choices. As one young man stated:

"If I made the choice to eat a pizza, I will not all of the sudden choose a salad. But if there would be two choices, a normal and a low-calorie version, then I would choose the latter".

Participants recommended school canteens as locations where marketing strategies that increase accessibility of low-calorie foods would be most effective. A majority perceived a shortage of those products in the assortment at school canteens currently. In contrast, participants contested the effectiveness of increasing accessibility of low-calorie choices in restaurants. The main argumentation for this finding concerned participants’ goal of eating out. Almost all described its purpose along the lines of ‘being away’ and ‘having fun’, thereby indicating that the caloric value of food choices was of less importance.

#### Perceived healthiness of low-calorie choices

Related to the importance of accessibility, participants stressed that the perceived healthiness of low-calorie choices plays a crucial role in the effectiveness of interventions as well. Some reported to have doubts regarding the healthiness of low-calorie choices. While all participants saw fruit and vegetables as healthy low-calorie choices, some were sceptical of the healthiness of other low-calorie products. Frequently mentioned products that induced this scepsis were light versions of soft drinks and pizzas. Participants perceived the additives and the ingredients that replace the sugar and the fat in light products to be unhealthy, and therefore less attractive. A young woman stated:

*“People think that a ’light’ pizza with 50*% *less fat is healthy. But if you look closely at the ingredients, they add a lot of other stuff. (…) We think ‘light’ means healthy, but I don’t think that’s always the case.”*

#### Identifiability of low-calorie choices

Another condition for the effectiveness of interventions that emerged was that they should make low-calorie choices evident to consumers. Participants regularly stressed the importance of clear nutritional information on food packages, especially for those who have insufficient knowledge of nutritional values. On-pack information should be clear enough for all consumers to make an informed decision. As a woman with a higher education said:

“You can’t expect consumers to be experts in every area. Even I have problems reading the product information. Packaging should give clear information on nutritional values.”

Participants showed a strong preference for the use of food labels that carry the traffic-light system to indicate nutritional values. Such colourful information would make identification of low-calorie choices easier. Some participants believed that the government should force food suppliers to provide food labels with the traffic-light system:

“I really like the use of colours. It’s very easy to use. When you see the red colour, you know it’s bad for you, and if it’s green then it’s good (…) This system should be mandatory.”

The quote above, given by a mother of two children, reveals that the perceived effectiveness of educational interventions was also associated with the ease-of-use of information. Some participants complained about the complexity of the information on product packages, which impedes the identification of healthy choices. Virtually all who addressed the ease-of-use of information agreed that educational interventions should force information to be simple, comparable, and uniform.

#### Perceived effectiveness of combinations of intervention strategies

In addition to the assessment of separate interventions, participants regularly discussed the effectiveness of combinations of interventions. Most thought that combining educational, marketing, and legal interventions would more effectively stimulate consumers to make low-calorie choices than each intervention separately. Some participants pointed at the success of books on dieting, thereby claiming that educational interventions will be more effective when they are combined with marketing strategies. Furthermore, virtually all thought that tax measures would be more effective if subsidies were provided at the same time:

“It would be good to not only make the bad things more expensive, but also to make the good things cheaper. That way it remains balanced. To me, that seems more effective.”

### Perceived fairness of intervention strategies for low-calorie food choices

Figure 2c shows the perceived fairness of the nine archetypical interventions. The following section describes the six aspects that can be discerned with respect to the perceived fairness of interventions.

#### Fairness of encouragement versus discouragement of choices

First, the distinction between encouragement and discouragement of choices appeared to be relevant for the perceived fairness of interventions. Participants regularly discussed whether interventions should focus on encouragement of low-calorie choices or on discouragement of high-calorie choices. Figure 2c indicates that perceptions of fairness not only varied across interventions, but also within interventions. Overall, encouraging strategies were rated slightly more fair than the discouraging counterparts (1st, 3rd, and 5th versus the 2nd, 4th, and 6th). When asked to account for the preference of encouraging strategies, a woman claimed:

“Rewarding healthy actions is fair. Interventions should be structured in a positive manner.”

In contrast, proponents of discouraging interventions often pointed at the effectiveness of discouragement to illustrate why they thought these interventions were fair. While arguing that taxes for high-calorie foods are a fair way of stimulating low-calorie choices, a formerly obese man stated:

“To make people think about and change their food choices, you have to hit them where it hurts to be effective: in their wallet.”

In addition, similar to the perception that combinations of interventions increase effectiveness, tax measures for high-calorie foods were also perceived fairer when they were paired with subsidies for low-calorie foods.

#### Societal fairness of intervention strategies

Second, to assess the fairness of interventions, participants took potential consequences for society into consideration. The perceived monetary costs and benefits of intervention strategies caused a differentiation in appraisal of fairness. A few participants questioned the fairness of governmental food-education campaigns. They claimed that these campaigns are not effective in combating the obesity problem and are therefore not an efficient use of community resources. A majority of participants, however, contested this view by claiming that a lack of knowledge lies at the heart of the obesity problem. They stressed that extensive food choice education is a fair way of stimulation; some even argued that it is an absolute necessity. Furthermore, those in favour of health campaigns emphasised the lower costs for healthcare when people would more often make low-calorie food choices.

The consequences of legal interventions were also much debated. Participants particularly addressed the effects of taxes and subsidies on society. While virtually all perceived subsidies to be a fair way to stimulate low-calorie food choices because they decrease consumers’ expenses, this was different for taxes. Those who thought taxing of high-calorie foods was fair argued that increasing the prices is profitable for the government, while it also stimulates consumers to make low-calorie choices more often. Opponents, in reaction, were suspicious of the use of the tax revenues and pointed at the conflicting roles the government plays with regard to legal interventions:

“The government says you shouldn’t smoke, but at the same time they expect to generate large amounts of money by immensely taxing the cigarettes. The taxing of food is exactly the same.”

#### Fairness of effects on consumer groups

Third, participants associated fairness with the implications that interventions have for specific groups of consumers. Many thought that excessive taxing of high-calorie foods would heavily burden people with a low budget. As a result, these people would have fewer resources available for participation in social happenings, sports, and other leisure activities. Participants therefore feared for social exclusion of that group, which they regarded as an unfair consequence of the intervention.

Children were another specific group that was addressed. Participants with children often voiced concerns regarding the aggressive marketing of unhealthy foods towards kids. Some therefore favoured a legal restriction of the promotion of unhealthy foods towards children. In contrast, these participants welcomed marketing and promotion when it concerned healthy foods like fruit and vegetables. As a result, a majority agreed that it would be fair to implement marketing and educational interventions to stimulate children to choose low-calorie foods. Some even stated that both teaching children about the origins of foods and providing information about healthy eating should be implemented in school programs.

#### Fairness of effects on the food industry

In addition to the effects on consumer groups, consequences for the food industry were also taken into account. Some participants felt that hindering the food industry too much with interventions would be unfair. They perceived legal interventions such as taxing and restricting advertising to be unfair if they threaten companies’ existence. During one interview, an elderly man felt that farmers would be unfairly cornered due to certain interventions:

“I have an agricultural background and I know how hard it is for farmers to make a living. Some legal interventions would make that even harder and that would not be fair.”

When talking about the effects of interventions on the food industry, participants also pointed at potential undesired side effects for consumers. Some feared that, as a result of mandatory traffic-light labeling, food manufacturers would manipulate food ingredients. This manipulation could compromise the healthiness of low-calorie products:

“A drawback of that intervention (traffic-light labeling) is that there is a danger of manipulation with ingredients. Food suppliers will do anything to get the red dots off their packages.”

#### Fairness of disseminated information

Fifth, participants often discussed the fairness of educational interventions. The accuracy of both presently existing ways of nutritional disclosure and the ones proposed by the interventions was heavily contested. The fairness of such interventions therefore was questioned. A large number of participants felt that food suppliers sometimes make inaccurate health claims with regard to their products. Identical sentiments were expressed with regard to the clarity of product content information. Participants regularly complained about the complexity of information on food packaging. Some displayed frustration towards food manufacturers by pointing at the numerous E-numbers (chemical additives) they put on the ingredient lists. Others participants pointed at unpronounceable names they encounter when reading information on food packages. Therefore all were in favour of more comprehensible information on food packages.

To increase the fairness of educational interventions, the majority of participants agreed that the source of such strategies needs to possess extensive knowledge of nutrition and health claims. In addition, the source of interventions should be autonomous and independent of the food industry. Because the current labels in the Netherlands were introduced by the food industry, many participants disapproved of existing food labeling systems.

#### Perceived liberty, autonomy, and privacy of food choice

Last, a large number of participants were afraid that interventions would threaten their liberty, autonomy, and privacy. The paternalistic nature of both taxing high-calorie foods and restricting high-calorie food advertising led to varying opinions regarding fairness. Participants who perceived themselves to be solely responsible for food choice thought that consumers should not be ‘told’ what is best for them because that would imply that they are incapable of making choices on their own. As one elderly man firmly stated:

“It is starting to look like a dictatorial situation. Discouraging all the unhealthy stuff and fill the streets with healthiness propaganda (…) If we can’t freely choose to enjoy unhealthy things, then our society is doomed.”

Others who did acknowledge the additional responsibility of other stakeholders viewed governmental participation as an important condition for the legitimacy of interventions. Those participants considered it fair to be patronised to a certain degree as long as they were not forced to make a certain choice:

“Whether or not you want to make unhealthy choices, it still is your own choice. And if these interventions navigate you to healthier choices without forcing you, then that’s good for everyone.”

Participants reached consensus on the fact that interventions should not threaten the freedom of choice. All stressed that being able to make the food choices you want without being restricted is a great good in a free society.

Related to the discussion about free choice was the issue of privacy. Participants considered interventions that register personal food choices to be an invasion of privacy. An employer tracking food choices in the worksite cafeteria to give feedback on the amount of calories employees buy was therefore seen as unfair:

“I don’t want my employer to know what and how much I eat and drink. That’s none of their business.”

#### Re-assessing acceptability

During the last part of the interviews and focus group discussions participants once more rated their acceptance of the interventions. Looking at differences between the first and second classification of acceptance in Figures 2d, it becomes apparent that making high-calorie choices more expensive became less acceptable on second thought. When the moderator asked why that intervention had become less acceptable, a wealthy woman stated:

“First I didn’t really have a problem with it. But I realised that to some others it’s just not fair to tax foods.”

Besides a noticeable change in acceptance of taxing high-calorie foods, participants reported no major shifts when comparing the first and second classifications of acceptability. Neutral ratings, however, had declined for the majority of the interventions during the second classification. Most participants who reported less neutral ratings classification attributed this difference to the exchange of ideas and opinions with others. The sharing of arguments for and against acceptance of interventions helped many to form an opinion. A man indicated:

“There are no big differences. And the things that did change, I did that because some good arguments were brought forward by others in this group.”

## Discussion

The current study confirms existing literature concerning the beliefs that influence consumer acceptance of intervention strategies [22]. Furthermore, the underlying aspects related to these beliefs are identified for interventions in the food domain. We show that both general beliefs regarding obesity and intervention-specific beliefs regarding the effectiveness and fairness are associated with consumer acceptance of interventions for low-calorie food choices. The general beliefs regarding obesity concern issues of responsibility for food choice and problem awareness. Intervention-specific beliefs, on the other hand, deal with statements about why interventions are (not) effective and why the interventions are (not) a fair way of stimulating low-calorie choices.

The majority of quotes that were given during the discussions on acceptability consisted of comments on the effectiveness and the fairness of interventions. In addition to this finding, participants showed no major differences between the classifications of acceptability in the beginning and at the end of the discussions. These two findings strengthen the claim that acceptability, perceived effectiveness, and perceived fairness are interrelated beliefs with regard to interventions in the food domain.

Participants were aware that obesity numbers have risen over the past three decades. The majority attributed this phenomenon to a food environment that encourages overeating, much like the objectively observed ‘obesogenic environment’ in literature [33]. This attribution explains the finding that a majority of participants thought most interventions were acceptable.

In addition, the perceived responsibility for food choice was related to the acceptance of interventions. While all study participants acknowledged a personal responsibility for their food choice, they did not agree on how much others, particularly the government and food suppliers, were also responsible. Those who thought such third parties were also responsible were more likely to show acceptance of interventions. These findings agree with Chambers and Traill [34] and Barry et al. [16], who found that the support for obesity prevention policies was greatest when causes for obesity rates were attributed to factors beyond individual control.

Literature on the effectiveness of interventions suggests that strategies that discourage high-calorie choices are more effective than strategies that encourage low-calorie choices [35,36]. Many participants, however, viewed interventions that encourage low-calorie choices to be more effective. To identify a rationale for this discrepancy, the relation between the perceived effectiveness and the actual effectiveness of interventions needs to be studied. For instance, it would be interesting to see what happens when evidence for effectiveness is provided, especially because positive statements regarding effectiveness seem to increase acceptance of interventions [37].

Legal interventions, and thus governmental involvement in interventions, remained a controversial issue throughout the discussions. This controversy appeared to be twofold. First, fairness classifications of legal interventions differed between participants. Second, the discussions showed a discrepancy between the perceived fairness and the perceived effectiveness of legal interventions. Even though roughly half of the participants perceived legal interventions to be unfair, many did see the government as the most capable source to intervene effectively. Experts believe that legal interventions and governmental involvement are indispensable when it comes to reducing obesity rates [38]. This sentiment has been echoed in many other studies [1,39,40]. To increase acceptance of governmental policies in the food domain, concerns about governmental involvement therefore need to be addressed.

Child-focused interventions show great promise in reducing childhood overweight and obesity [41]. Participants expressed similar opinions by emphasizing the importance of child-focused interventions. Many thought that educational and marketing interventions both at schools and at home were acceptable and effective strategies to stimulate children to choose low-calorie foods. Participants saw parents as the most appropriate source to implement interventions for their children because they have both the responsibility and the opportunity to stimulate children to choose low-calorie foods. This finding is similar to Mitchell et al. [42], who stress the importance of interventions aimed at parents to improve children’s eating behaviour.

With regard to the generalizability of the findings, two issues are worth mentioning. First, the results merely reflect the viewpoints of a sample of Dutch consumers who varied with regard to age, gender, and income. It is possible that variations in political and cultural circumstances lead to different levels of acceptance as well as other mechanisms underlying acceptance. For instance, Mazzocchi et al. [43] found differing levels of acceptance of obesity prevention policies across five European countries, presumably due to the familiarity with specific policies. However, with respect to the mechanisms underlying acceptance, we think that the concepts that we identified are quite generalizable, particularly because these also underlie acceptance of interventions in non-food domains [22].

Second, this study limited its scope to acceptance of interventions for consumer choices in terms of caloric value. It would be extremely useful to see whether consumers’ perceptions are construed similarly when talking about interventions that target other product characteristics, e.g. increased fruit consumption or decreased salt consumption. Similar to the expected generalizability of the concepts underlying acceptance across cultures, we expect them to be generalizable to acceptance of interventions aimed at other nutritional characteristics as well.

With respect to the methods that were used, again two issues are worth mentioning. First, a potential limitation is that a small number of intervention strategies were used to assess consumer acceptance. However, the interventions were carefully selected on the basis of social marketing literature [28] to represent different types of interventions (education, marketing, and law). The use of nine archetypes also facilitated the possibility of exploring participants’ acceptance of these generic interventions as well as more detailed versions of these interventions (e.g. specific locations and sources). Furthermore, participants could have experienced confusion when a larger number of interventions would have been used.

Second, one can argue that the order of the different parts of the discussions led to modifications in participants’ ratings of acceptance. Because this sequence was not varied, potential biases, particularly order effects [44] and belief overkill [45], cannot be ruled out. Looking at participants’ argumentations for their ratings, we have little reason to believe that these side effects indeed surfaced and compromised the legitimacy of participants’ responses. Furthermore, the specific order was employed to prevent that discussions on effectiveness and fairness of interventions would influence the initial rating and discussion on acceptance. This enabled us to see whether statements about effectiveness and fairness would surface spontaneously, like they did.

An important last note is that our research emphasises interventions that focus on calorie intake rather than calorie expenditure. We chose this emphasis because literature stresses that curtailment of calorie intake is of greater importance to reduce obesity rates [5]. That does not mean, however, that interventions that target calorie expenditure should receive less attention, particularly because those kinds of interventions also show great promise in reducing obesity rates [46]. Future research concerning consumer acceptance of interventions for obesity prevention should therefore also include interventions that emphasise calorie expenditure.

## Conclusions

Policymakers in the food domain need to be able to anticipate consumer acceptance of intervention strategies. Knowledge of factors that influence consumer acceptance therefore is crucial. The present study identifies the beliefs and underlying aspects that influence acceptance of interventions from a consumer perspective. The findings can be used by policymakers to anticipate consumer reactance towards interventions and to negotiate the development and communication of new strategies.

The present article enriches existing literature on food choice interventions in two ways. First, it confirms research on the beliefs that influence acceptance of interventions from other domains. Second, and most important, it identifies the underlying aspects of these beliefs specifically for food choice interventions. Besides consensus on a few issues, the outcomes show that consumers’ classifications of acceptance, perceived effectiveness, and perceived fairness of interventions differ between and within individuals. Logical next steps would be to segment people based on how they perceive specific interventions and to explore how these segments should be approached to increase acceptance. This will enable us, for instance, to see whether providing evidence for actual effectiveness of interventions will increase the perceived effectiveness and subsequently the acceptance of interventions.

## Competing interest

The authors declare that they have no competing interests.

## Authors’ contributions

CB carried out literature research, conducted the interviews and focus groups, analysed the data, and contributed significantly to both the design of the interview and focus group guide and the drafting of the manuscript. IL contributed substantially to the design of the interview and focus group guide and has been continuously involved in drafting the manuscript. FR and HT contributed substantially to the design of the interview and focus group guide and helped drafting the manuscript. All authors read and approved the final manuscript.

## Pre-publication history

The pre-publication history for this paper can be accessed here:

http://www.biomedcentral.com/1471-2458/13/1073/prepub
